# Safe, One-Pot, Homogeneous Direct Synthesis of H_2_O_2_

**DOI:** 10.1021/jacs.2c13149

**Published:** 2023-02-17

**Authors:** Seiji Ogo, Takeshi Yatabe, Tamon Tome, Riko Takenaka, Yoshihito Shiota, Kenji Kato

**Affiliations:** † Department of Chemistry and Biochemistry, Graduate School of Engineering, Kyushu University, 744 Moto-oka, Nishi-ku, Fukuoka 819-0395, Japan; ‡ International Institute for Carbon-Neutral Energy Research (WPI Academy I2CNER), Kyushu University, 744 Moto-oka, Nishi-ku, Fukuoka 819-0395, Japan; § Center for Small Molecule Energy, Kyushu University, 744 Moto-oka, Nishi-ku, Fukuoka 819-0395, Japan; ∥ Institute for Materials Chemistry and Engineering, Kyushu University, 744 Moto-oka, Nishi-ku, Fukuoka 819-0395, Japan; ⊥ Mitsubishi Gas Chemical Company Inc., Tokyo 100-8324, Japan

## Abstract

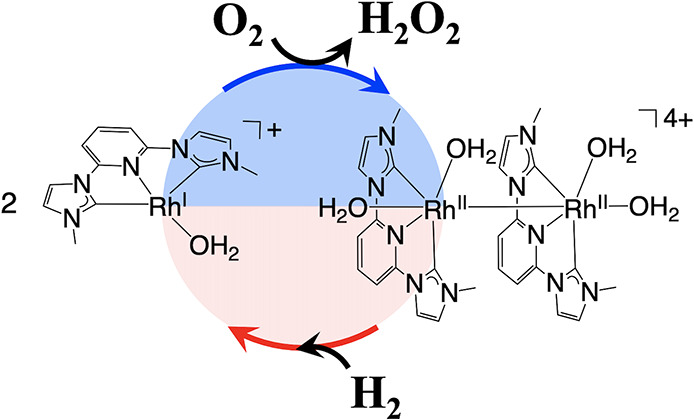

Hydrogen peroxide is an environmentally friendly oxidizing agent but current synthetic methods are wasteful. This is a result of the high flammability of H_2_/O_2_ mixtures and/or the requirement for cocatalysts. In this paper, we report the synthesis of H_2_O_2_ by means of a homogeneous catalyst, which allows a safe, one-pot synthesis in water, using only H_2_ and O_2_. This catalyst is capable of removing electrons from H_2_, storing them for the reduction of O_2_, and then permitting the protonation of the reduced oxygen to H_2_O_2_. The turnover number (TON) is 910 under an H_2_/O_2_ (95/5) atmosphere (1.9 MPa) for 12 h at 23 °C, which is the highest of any homogeneous catalyst. Furthermore, we propose a reaction mechanism based on two crystal structures.

Hydrogen peroxide (H_2_O_2_) is an important oxidizing agent but current synthetic methods are wasteful.^1−5^ This is because there is only a narrow range of H_2_/O_2_ mixtures that are not dangerously flammable, and the two gases must remain well-separated on-site.^6−9^ Furthermore, current methods often require diluents, cocatalysts, and/or organic solvents that must then be separated from the product stream and disposed of.^10−15^

If chemists were granted three wishes, they might desire (1) a safe reaction mixture, outside the flammability limit; (2) reaction in one vessel, without the need for transfer and separation; and (3) direct synthesis using only H_2_ and O_2_ (Table 1).

**Table 1:**
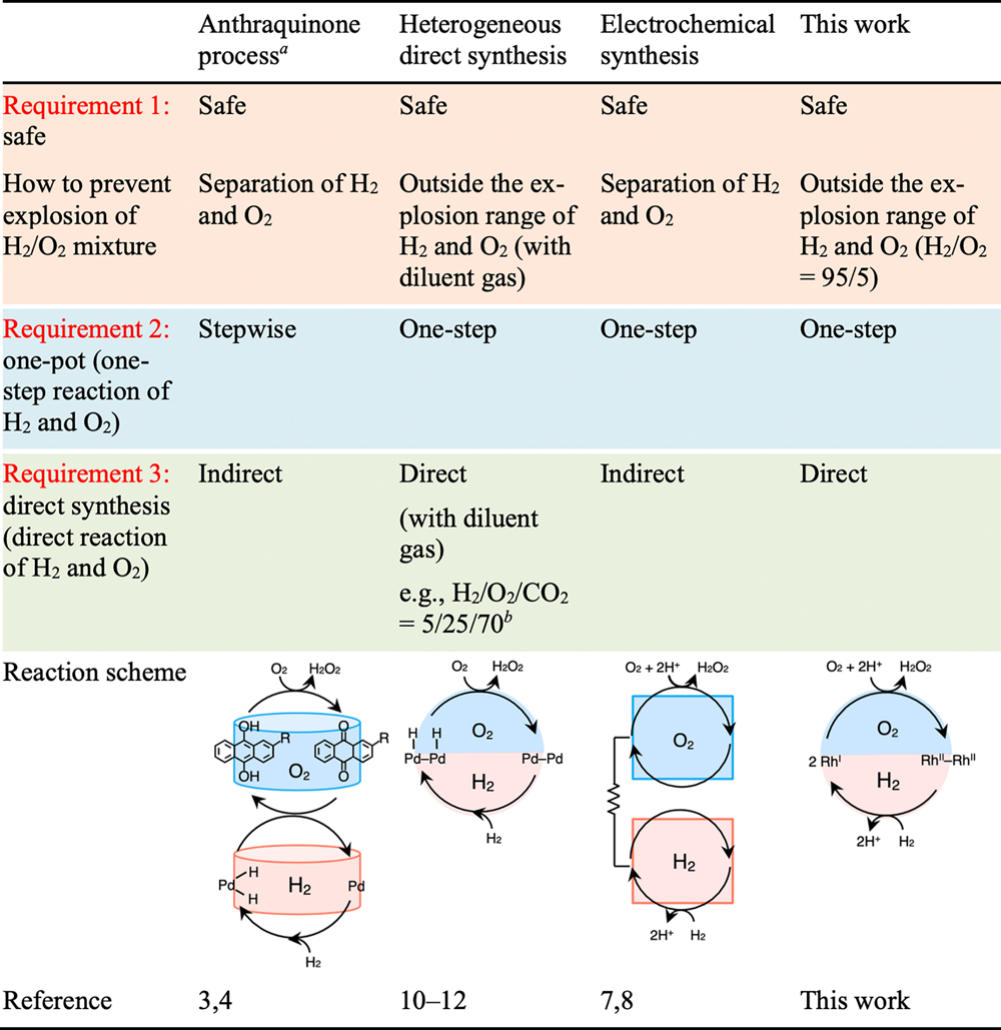
H_2_O_2_ Synthesis under Safe Conditions

a See Table S2 in the Supporting Information for a detailed comparison of the anthraquinone process and this work.

b See ref (10).

An example of just such a synthesis is thought to be conducted by extremophile microorganisms in nature.^16−18^ These organisms use hydrogenase enzymes that are usually degraded by O_2_, but it has been proposed that O_2_-tolerant hydrogenases might transfer electrons from H_2_ to O_2_, thereby reducing it to H_2_O_2_.

Inspired by this idea, we previously synthesized a complex with an iMP ligand {iMP = 2,6-bis(2-imidazolyl-1-methyl)pyridine} that stores electrons from H_2_ and, using a mixed gas of H_2_ and O_2_ outside the explosion range, investigated the direct synthesis of H_2_O_2_ in one flask.^19^ However, the reactivity of this electron storage complex with O_2_ was very low, and H_2_O_2_ was hardly produced catalytically. We synthesized a Rh^III^_2_ peroxide complex stepwise by irradiating a Rh^II^_2_ complex and then showed the liberation of small amounts of H_2_O_2_.

The ligand of the desired catalyst requires electron-withdrawing properties to oxidize H_2_, but electron-donating properties to reduce O_2_. We have now improved the catalyst with a strongly electron-donating N-heterocyclic carbene ligand in order to catalytically reduce O_2_ without irradiation and, in this paper, describe its behavior and properties. We report how the catalyst allows the homogeneous synthesis of H_2_O_2_ starting with extracting electrons from H_2_, then using those electrons to reduce O_2_, and finally, acquiring protons from water to release the final product and return to the starting state.

This is a homogeneous H_2_O_2_ synthesis that meets the above three requirements: outside the flammability limit of the mixed gas (H_2_/O_2_ = 95/5),^9^ one flask, and homogeneous direct synthesis. The reaction mechanism of the homogeneous synthesis is discussed, starting from the structure of the Rh^II^_2_ complex (**1**) that reacts with H_2_, then the structure of the Rh^I^ complex (**2**) that reacts with O_2_.

The initial rhodium dimer complex [Rh^II^_2_(L)_2_(OH_2_)_4_](NO_3_)_4_ {[**1**](NO_3_)_4_, L = 2,6-bis(1-methylimidazol-2-ylidene)pyridine} was prepared from the reaction of a Rh^III^ synthetic precursor [Rh^III^(L)(OH_2_)_3_](NO_3_)_3_ {[**3**](NO_3_)_3_} with H_2_ followed by oxygenation by O_2_ at 23 °C in water. Characterization of **1** was performed by ^1^H NMR and ^13^C NMR spectroscopies (Figures S4 and S5), C–H correlation spectroscopy (COSY) (Figure S6), ultraviolet–visible–near-infrared (UV–vis–NIR) absorption spectroscopy (Figure S7), X-ray photoelectron spectroscopy (XPS, Figure S8c), and elemental analysis. Single crystals of CH_3_CN-coordinated **1** [Rh^II^_2_(L)_2_(OH_2_)_2_(CH_3_CN)_2_](CF_3_SO_3_)_4_ {[**4**](CF_3_SO_3_)_4_} were obtained from the slow evaporation of a CH_3_CN/H_2_O solution of **1** after replacing the NO_3_^–^ ions with CF_3_SO_3_^–^ ions. An ORTEP drawing shows that the two Rh metal centers adopt a distorted-octahedral geometry and are linked by a metal–metal bond to form the dimer structure (Figure 1). The Rh–Rh distance {2.7378(4) Å} of **4** was longer than those of other unsupported Rh^II^ dimer complexes {2.624(1)–2.7052(5) Å} (Table S3).^19−21^ A ^1^H NMR spectrum shows peaks in the diamagnetic region, which suggests that each Rh^II^ metal center is linked by a metal–metal bond (Figure S4). A UV–vis–NIR absorption spectrum of **1** shows a shoulder at 380 nm, which is similar to the spectrum of our previous Rh^II^ dimer with the iMP ligand (Figure S7).^19^ The XPS spectrum of **1** exhibits Rh 3d_3/2_ and 3d_5/2_ peaks at 313.3 and 308.7 eV (Figure S8c), which are similar to those of our previously reported Rh^II^ dimer complex and are lower than those of the related Rh^III^ complex **3**.^19^ These results, taken together, indicate that complex **1** adopts a dinuclear structure with divalent Rh centers.

**Figure 1:**
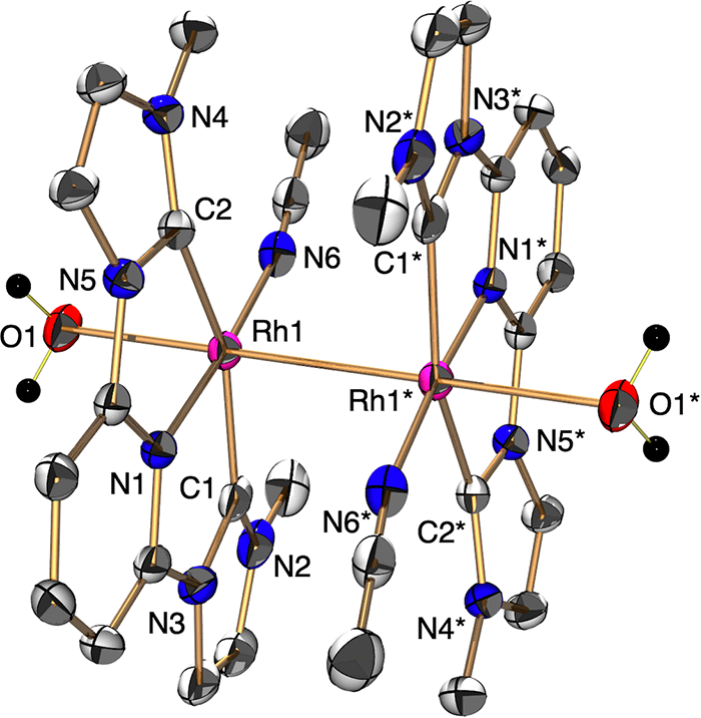
An ORTEP drawing of CH_3_CN-coordinated **1** [Rh^II^_2_(L)_2_(OH_2_)_2_(CH_3_CN)_2_](CF_3_SO_3_)_4_ {[**4**](CF_3_SO_3_)_4_} with the ellipsoids at 50% probability. Counteranions (CF_3_SO_3_^–^) and hydrogen atoms (except for the aqueous ligands) are omitted for clarity.

Complex **1** reacts with H_2_ to form a low-valent Rh^I^ complex [Rh^I^(L)(OH_2_)](NO_3_) {[**2**](NO_3_)} in water (eq 1). We characterized this complex by UV–vis–NIR absorption spectroscopy (Figure S9), XPS (Figure S8a), ^1^H NMR spectroscopy (Figure S10), and elemental analysis. A UV–vis–NIR absorption spectrum of **2** shows a broad absorption band at 650–1000 nm with/without CH_3_COONa (Figure S9), which is likely to arise from a charge transfer band, derived from metal–metal interactions, as seen in Rh^I^ polypyridyl complexes.^25−30^ An XPS spectrum of **2** shows Rh 3d_3/2_ and 3d_5/2_ peaks at 312.2 and 307.5 eV, respectively (Figure S8a). These binding energies were lower than those of Rh^II^ complex **1** (313.3 and 308.7 eV) (Figure S8c), but similar to those of the reported Rh^I^ complexes.^19,28−32^ Since the O_2_-sensitive **2** could not be crystallized, its structure was confirmed by X-ray analysis as a CO-adduct of **2**, [Rh^I^(L)(CO)](NO_3_) {[**5**](NO_3_)} (Figure S11). An ORTEP drawing of **5** shows that the Rh metal center has a square planar structure composed of L and the CO ligand. This geometry has been observed for other Rh^I^ complexes.^19,22−24^

These results indicate that complex **1** extracts two electrons from H_2_ to produce Rh^I^ complex **2** and that this complex is stabilized by the electron-withdrawing properties of L.
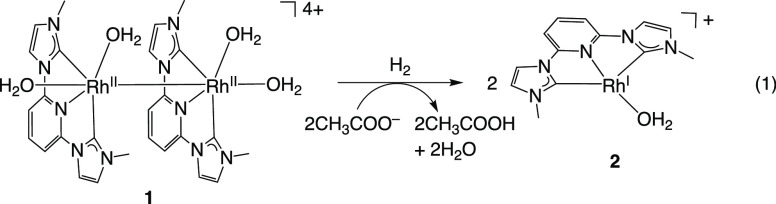
1

Our next step was to investigate the recovery of **1** by the reaction of **2** with O_2_ under dark conditions (eq 2), with the reaction being monitored by UV–vis–NIR absorption spectroscopy (Figure S14). Injection of O_2_ gas into an aqueous solution of **2** led to the disappearance of the absorption bands at long wavelengths and the formation of a shoulder at around 380 nm with/without CH_3_COONa (Figure S14). The absorption spectrum of this reaction solution is similar to that of **1** (Figure S7).
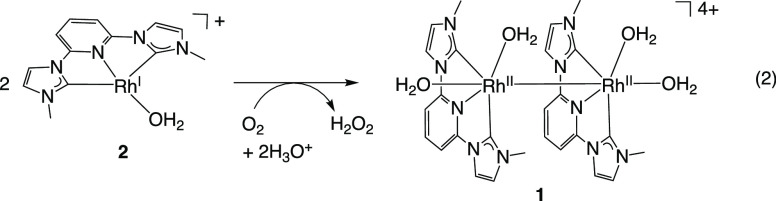
2

Quantitative analysis of the formed H_2_O_2_ was conducted by iodometric spectrophotometry (Figure S15), using NaI as the redox indicator.^33^ In order to separate the catalyst and product, after removing the Rh complexes by changing the counterion from NO_3_^–^ to BPh_4_^–^ and filtering, an excess of NaI was added to the remaining reaction solution in the presence of O_2_. The solution showed an absorption band at 353 nm, assigned to the absorption band of I_3_^–^ generated from the two-electron oxidation of 3I^–^ by H_2_O_2_ (Figure S15). The yield of H_2_O_2_ was determined as 53% based on the amount of I_3_^–^ calculated using the absorbance at 353 nm (ε = 2.4 × 10^4^ M^–1^ cm^–1^ for I_3_^–^ in H_2_O).^33^

Quantitative analysis of the formed H_2_O_2_ was also conducted by titration with oxo[5,10,15,20-tetra(4-pyridyl)porphyrinato]titanium(IV) complex (Ti-TPyP) (Figure S16).^33,34^ The UV–vis absorption spectrum of the following mixture was obtained: an aqueous HCl solution of Ti-TPyP, a reaction solution of **2** in the presence of O_2_ and an aqueous HClO_4_ solution. An absorption band at 433 nm was decreased compared with that of the control experiment, indicating the formation of a Ti peroxide species by the reaction of the Ti-TPyP reagent with H_2_O_2_. The yield of H_2_O_2_ was determined as 52% without CH_3_COONa (50% with CH_3_COONa) based on a calibration curve obtained from a standard H_2_O_2_ solution (Figure S16). The produced H_2_O_2_ decomposed less than 10% into H_2_O under stoichiometric and catalytic conditions, which was confirmed by the isotope labeling experiment using H_2_^18^O_2_ (Figure S17).

The full catalytic cycle was performed with an aqueous CH_3_COONa solution of **1** (100 μM) under an H_2_/O_2_ (95/5) atmosphere (0.5–1.9 MPa) at 23 °C for 12 h under dark conditions–this is a nonexplosive gas mixture (Figure 2).^9^ The produced H_2_O_2_ was quantified by the Ti-TPyP reaction method. The turnover numbers (TONs) were increased depending on the total pressure of H_2_/O_2_ gas. The maximum TON was determined to be 910 under an H_2_/O_2_ (95/5) atmosphere (1.9 MPa) for 12 h at 23 °C, which is the highest TON in the direct synthesis of H_2_O_2_ from H_2_ and O_2_ using a homogeneous catalyst (Table 2). The initial turnover frequency of the catalytic reaction under an H_2_/O_2_ (95/5) atmosphere (1.9 MPa) for the first 1 h at 23 °C is 164 h^–1^ (163 mol kg_cat_^–1^ h^–1^), which is comparable to those of heterogeneous systems (60.8–180 mol kg_cat_^–1^ h^–1^).^8^ No H_2_O_2_ was formed without **1**, H_2_, or O_2_.

**Figure 2:**
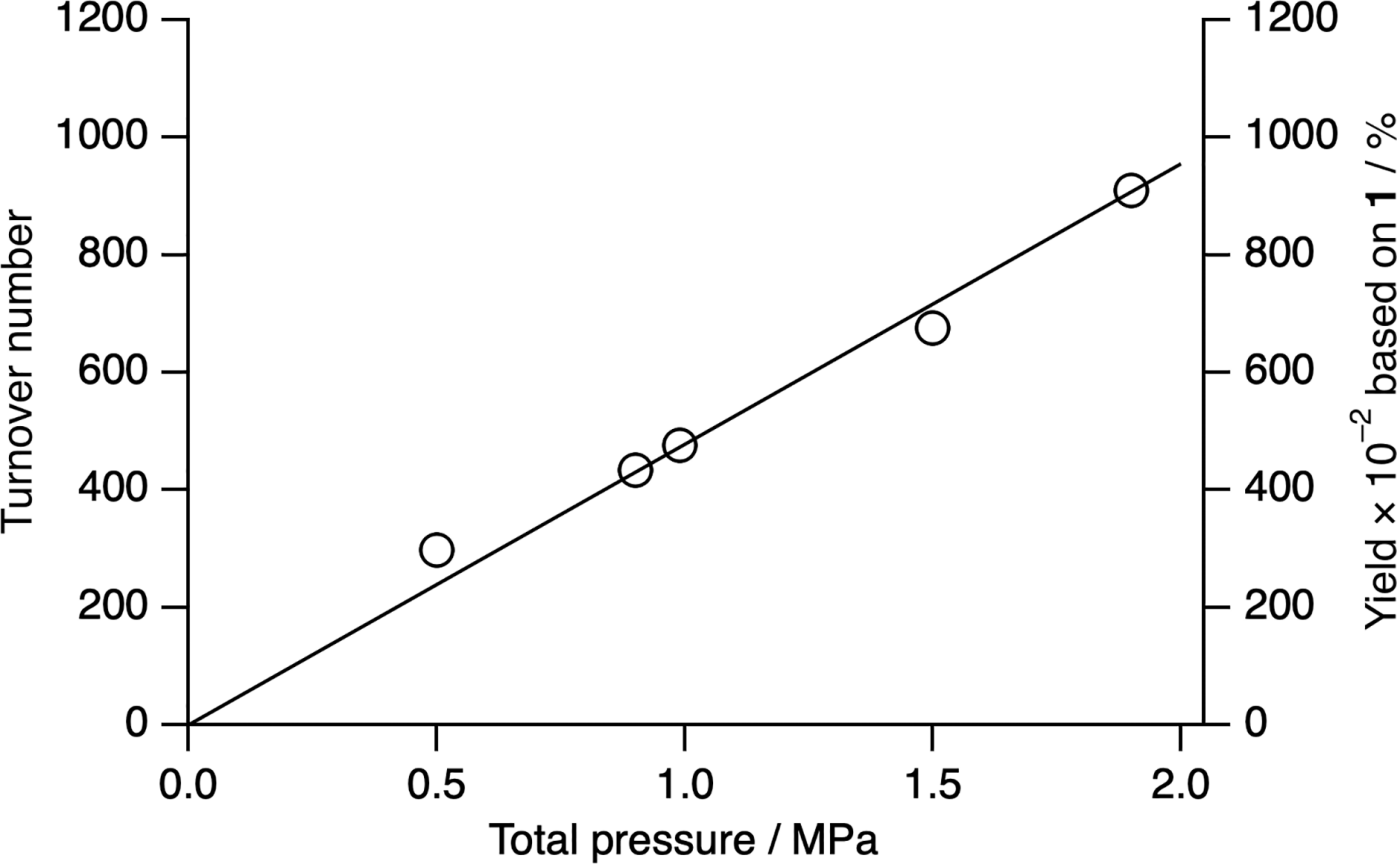
Pressure-dependent H_2_O_2_ production catalyzed by **1** under an H_2_/O_2_ (95/5) atmosphere for 12 h at 23 °C. The maximum TON is 910 under an H_2_/O_2_ (95/5) atmosphere (1.9 MPa).

**Table 2:** Requirements of Homogeneous H_2_O_2_ Synthesis

Requirement		Previous work^a^	This work
Safe	H_2_ (%)	95	95
	O_2_ (%)	5	5
	Temperature (°C)	40	23
	Solvent	Water	Water
One-pot	Reaction vessel	High-pressure glass cylinder	High-pressure glass cylinder
Direct synthesis	H_2_ (MPa)	0.76	0.475–1.8
	O_2_ (MPa)	0.04	0.025–0.1
	TON	3.8	910
	Catalyst (mmol %)	0.11	0.22
	Reactivity of Rh^I^ species to O_2_	Rh^I^ species hardly reacts with O_2_	Rh^I^ species reacts with O_2_

a See ref (19).

The initial rate of H_2_O_2_ formation under catalytic conditions was investigated by varying the concentration of **1** at an H_2_/O_2_ (95/5) pressure of 1.9 MPa at 23 °C (Figure S18). It exhibits first-order kinetics, implying that the rate-determining step is the reaction of **1** with H_2_. A catalytic reaction using the in situ generated **2** under an H_2_/O_2_ (95/5) atmosphere (1.9 MPa) at 23 °C for 12 h under dark conditions gives TON as 908, suggesting complex **2** is part of the catalytic cycle. Under stoichiometric conditions, the pH was 4.9 with CH_3_COONa and 3.9 without CH_3_COONa. Under catalytic conditions, the pH was 8.3 with CH_3_COONa (0.5 M) and 3.9 without CH_3_COONa. The effect of CH_3_COONa on the formation of **1** and **2** was investigated by UV–vis absorption spectroscopy and ^1^H NMR spectroscopy, resulting in acetate ions interacting with **1** or **2** in a 1:1 or 1:2 ratio, respectively (Figures S4b and S19).

Qualitative analysis of H_2_O_2_ formed from the catalytic reaction was conducted with gas chromatography–mass spectrometry (GC–MS, Figure S20). The GC mass spectrum of the catalytic reaction solution, with Rh species removed, showed a signal at *m*/*z* = 34 assigned to H_2_O_2_ (Figure S20a). In an isotope labeling experiment using ^18^O_2_, this signal shifted to *m*/*z* = 38 (Figure S20c). During the catalytic reaction by **1**, no metal nanoparticles were formed, as confirmed by a dynamic light scattering measurement. These results indicate that complex **1** catalyzes the synthesis of H_2_O_2_ as a single catalyst, even under a nonexplosive H_2_/O_2_ gas mixture.

The electrochemical properties of [**3**](NO_3_)_3_ and an analogue with the iMP ligand, [Rh^III^(iMP)(OH_2_)_3_](NO_3_)_3_ {[**6**](NO_3_)_3_}, were investigated by differential pulse voltammetry in an aqueous solution of CH_3_COONa (0.1 M) (Figure S21). The cathodic peak at −0.324 V versus Ag/AgCl for **3** is more negative than that for **6** (−0.164 V versus Ag/AgCl). This also shows that the electron-donating ability of L is much stronger than that of iMP.

Based on the above results, we propose the following catalytic reaction mechanism (Figure 3): First, Rh^II^ dimer complex **1** extracts electrons from H_2_ to form the electron storage catalyst **2**. This step is aided by CH_3_COO^–^ behaving as a Lewis base to abstract the protons and leave the electrons behind. Complex **2** then transfers two electrons to O_2_ to produce H_2_O_2_.

**Figure 3:**
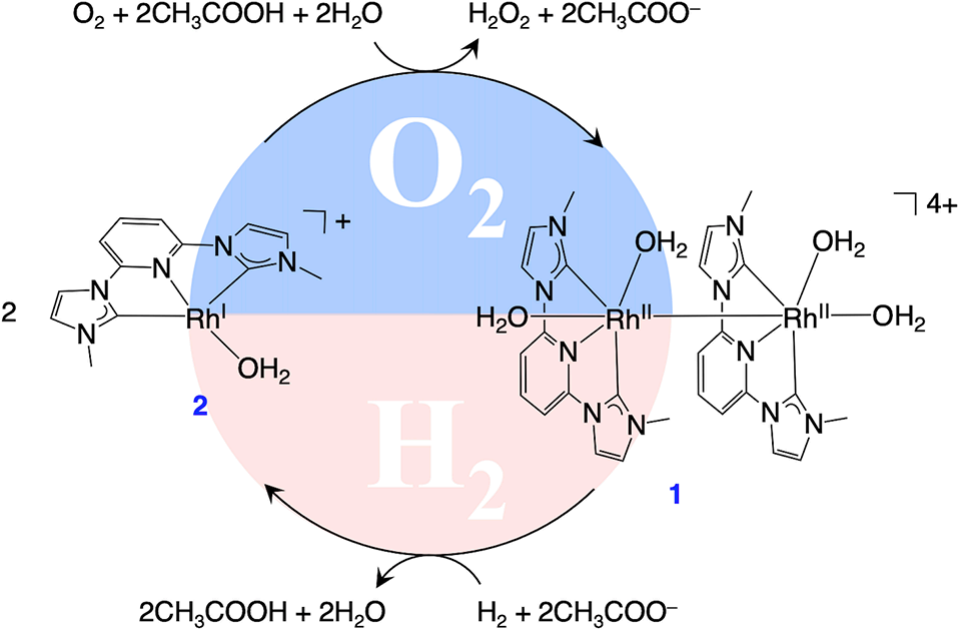
Proposed catalytic reaction mechanism of a synthesis of H_2_O_2_ from H_2_ and O_2_ using **1** as a catalyst in water. Acetate ions could replace with aqua ligands, but this has no apparent effect on the catalytic mechanism.

In conclusion, we have synthesized a complex that stores electrons from hydrogen and transfers them to oxygen, thereby catalyzing the oxidation of the former and the reduction of the latter, in a safe, one-pot, aqueous process. The TON is the highest of any homogeneous catalyst. We are confident that our system points the way to all round savings in cost and materials, once the simplicity of the process is taken into account.

## Supporting Information Available

Experimental details, characterization data of Rh complexes **1**, **2**, **3**, **4**, and **5** including NMR spectra, UV–vis–NIR absorption spectra, XPS spectra, an ORTEP drawing, and electrospray ionization (ESI) mass spectrum, and other experimental results including GC mass spectra, and differential pulse voltammograms (PDF)
