# Supplementary material for: Safe, One-Pot, Homogeneous Direct Synthesis of H2O2
Source: J Am Chem Soc. 2023 Feb 17;145(8):4384–8. doi: 10.1021/jacs.2c13149 (PMC9983002; doi:10.1021/jacs.2c13149)
Supplement: Supplementary file 1 — ja2c13149_si_001.pdf [file ja2c13149_si_001.pdf]

Supporting Information for

Safe, One-Pot, Homogeneous Direct Synthesis of H_2O_2

Seiji Ogo,* Takeshi Yatabe, Tamon Tome, Riko Takenaka, Yoshihito Shiota, Kenji Kato

*To whom correspondence should be addressed.

E-mail: ogo.seiji.872@m.kyushu-u.ac.jp

This PDF file includes:

Materials and Methods
Figures S1 to S21
Tables S1 to S3
References

A Table of Contents

Materials and Methods	page 1
Table S1	page 7
Table S2	page 8
Table S3	page 9
Figure S1	page 10
Figure S2	page 11
Figure S3	page 12
Figure S4	page 13
Figure S5	page 14
Figure S6	page 15
Figure S7	page 16
Figure S8	page 17
Figure S9	page 18
Figure S10	page 19
Figure S11	page 20
Figure S12	page 21
Figure S13	page 22
Figure S14	page 23
Figure S15	page 24
Figure S16	page 25
Figure S17	page 26
Figure S18	page 27
Figure S19	page 28
Figure S20	page 29
Figure S21	page 30
References	page 31

Experimental Section

Materials and Methods. All experiments were carried out under an N_2 atmosphere using standard Schlenk techniques and a glovebox. H_2O_2 formation reactions were carried out under dark conditions. H_2 (99.9999%) and O_2 (99.9999%) were purchased from Sumitomo Seika Chemical Co., Ltd., silver nitrate, ultra-pure water, sodium iodide, and sodium acetate were purchased from FUJIFILM Wako Pure Chemical Corporation, oxo[5,10,15,20-tetra(4-pyridyl)porphyrinato]titanium(IV) (Ti-TPyP) and CD_3CN were purchased from Tokyo Chemical Industry Corporation, H_2O_2 (30% aqueous solution) and $H_2{}^{18}O_2$ (90% ^{18}O-enriched, 2-3% in $H_2{}^{16}O$) were purchased from Sigma-Aldrich, $H_2{}^{18}O$ (98 atom%) was purchased from Taiyo Nippon Sanso Co., $^{18}O_2$ (98 atm%) was purchased from SI Science Co., Ltd., and D_2O and *N*,*N*-dimethylformamide (DMF)-d_7 were purchased from Cambridge Isotope Laboratories, Inc. These reagents were used without further purification. Rh^{III} tribromo complex $[Rh^{III}(L)(Br)_3]$ {L = 2,6-bis(1-methylimidazol-2-ylidene)pyridine} and $[Rh^{III}(iMP)(OH_2)_3](NO_3)_3$ {[**6**]$(NO_3)_3$, iMP = 2,6-bis(2-imidazolyl-1-methyl)pyridine} were prepared by the method described in the literature.[1,2]

Electrospray ionization-mass spectrometry (ESI-MS) data were obtained by a JEOL JMS-T100LC AccuTOF. ^1H and ^{13}C NMR spectra and C–H correlation spectroscopy (COSY) spectra were recorded on a Bruker Avance III 600 FT-NMR spectrometer. Chemical shifts were referenced to 3-(trimethylsilyl)propionic-2,2,3,3-d_4 acid sodium salt (TSP) in D_2O (^1H: 0.00 ppm, ^{13}C: 0.00 ppm) and protio solvent impurities in DMF-d_7 (^1H: 2.75 and 2.92 ppm) and in CD_3CN (^1H: 1.94 ppm) for ^1H NMR and ^{13}C NMR. Ultraviolet-visible-near infrared (UV-vis-NIR) absorption spectra were recorded on a JASCO V-670 UV-visible-NIR spectrophotometer. Gas chromatography-mass spectrometry (GC-MS) data were recorded on an Agilent 7890B GC coupled to a 5977B MSD and a SHIMADZU GCMS-QP 2010. The mass spectrometers were operated in the EI positive mode. X-ray photoelectron spectroscopy (XPS) spectra were recorded on an ULVAC PHI 5000 VersaProbe II system with an Al anode X-ray source. Binding energies were calibrated by the C 1s peak of carbon at 284.5 eV.[3] Dynamic light scattering (DLS) analysis was measured by using a Zetasizer NanoZSP (Malvern Instruments). All catalytic reactions were carried out in a Hiper glass cylinder (95 mL, TAIATSU TECHNO) or in a Parr Bench Top Micro Reactor system with 50 cm^3 cylinder volume.

[Rh^{III}(L)(OH$_2$)$_3$](NO$_3$)$_3$ {[3](NO$_3$)$_3$}. [Rh^{III}(L)(Br)$_3$] (150 mg, 0.258 mmol) and $AgNO_3$ (136 mg, 0.801 mmol) were stirred at 65 °C for 8 h in water/acetone (3/1) (80 mL) under dark conditions. After cooling to 23 °C, the precipitates were removed by filtration. The solvent of resulting solution was evaporated to yield a brown residue, which was purified by a Sephadex LH-20 column eluted with methanol to remove Ag salts. Water (2 mL) was added to the filtrate, and the solvent was removed by evaporation to afford a brown powder {yield 97% based on [Rh^{III}(L)(Br)$_3$]}. An XPS measurement of the product was carried out to confirm that no Ag salts were contained in the product. ^{1}H NMR (600 MHz, in D_2O, referenced to TSP): δ 4.37 (s, 6H, CH_3), 7.73 (s, 2H, NCH), 7.95 (d, 2H, pyridine-H), 8.33 (s, 2H, NCH), 8.51 (t, 1H, pyridine-H). ^{13}C NMR (150 MHz, in D_2O, referenced to TSP): δ 39.6 (NCH$_3$), 113.0 (pyridine-C), 122.5 (NCCN), 129.5 (NCCN), 150.5 (pyridine-C), 156.3 (pyridine-C), 172.9 (d, NCN). Anal. Calcd for {[**3**](NO$_3$)$_3$–1.5H$_2$O+1.5CH$_3$OH}: $C_{14.5}H_{22}N_8O_{12}Rh$: C, 28.87; H, 3.68; N, 18.57%. Found: C, 29.02; H, 3.49; N, 18.26%.

[Rh$^{II}_2$(L)$_2$(OH$_2$)$_4$](NO$_3$)$_4$ {[1](NO$_3$)$_4$}. An aqueous solution of [**3**](NO$_3$)$_3$ (7 mM, 10 mL) was stirred for 5 h at 23 °C under an H_2 atmosphere (0.3 MPa). After removing H_2, the resulting solution was stirred for 20 min under an O_2 atmosphere (0.1 MPa) under dark conditions. The solvent of the resulting solution was evaporated to yield dark-brown solid {yield 91% based on [**3**](NO$_3$)$_3$}. ^{1}H NMR (600 MHz, in D_2O, referenced to TSP): δ 3.99 (s, 12H, CH_3), 7.43 (br, 4H, pyridine-H), 7.55 (s, 4H, NCH), 7.93 (s, 4H, NCH), 8.11 (br, 2H, pyridine-H). ^{13}C NMR (150 MHz, in D_2O, referenced to TSP): δ 39.4 (NCH$_3$), 110.8 (pyridine-C), 121.6 (NCCN), 129.1 (NCCN), 147.3 (pyridine-C), 155.0 (pyridine-C), 183.8 (d, NCN). Anal. Calcd for [**1**](NO$_3$)$_4$·H$_2$O: $C_{26}H_{36}N_{14}O_{17}Rh_2$: C, 30.54; H, 3.55; N, 19.18%. Found: C, 30.20; H, 3.15; N, 19.41%.

[Rh^I(L)(OH$_2$)](CF$_3$SO$_3$) {[2](CF$_3$SO$_3$)}. Method A: An aqueous solution (30 mL) of [**3**](NO$_3$)$_3$ (30 mg, 52 μmol) was stirred at 23 °C for 5 h under an H_2 atmosphere (0.3 MPa) to form [**2**](NO$_3$). After removing H_2, an aqueous solution of $NaCF_3SO_3$ (89 mg, 0.52 mmol) was added to the resulting solution to yield the dark-green precipitates, which were collected by filtration and dried under reduced pressure {yield 84% based on [**3**](NO$_3$)$_3$}. Method B: An aqueous solution (5.0 mL) of [**1**](NO$_3$)$_4$ (25 mg, 25 μmol) was stirred at 23 °C for 3 h under an H_2 atmosphere (0.3 MPa). The pH of the reaction solution was decreased from 3.5 to 2.4 due to the release of H^+ from H_2. After removing H_2, an aqueous solution of $NaCF_3SO_3$ (43 mg, 0.25 mmol) was added to the resulting solution to form dark-green precipitates,

which were collected by filtration and dried under reduced pressure {yield 86% based on [**1**](NO_3)$_4$}.^{1}H NMR (600 MHz, in CD_3CN, referenced to protio solvent impurities): δ 3.52 (br, 6H, C*H*$_3$), 7.02 (br, 2H, pyridine-*H*), 7.11 (s, 2H, NC*H*), 7.51 (s, 2H, NC*H*), 7.87 (br, 1H, pyridine-*H*). Anal. Calcd for {[**2**](CF_3SO_3)–0.5H_2O}: $C_{14}H_{14}F_3N_5O_{3.5}RhS$: C, 33.61; H, 2.82; N, 14.00%. Found: C, 33.53; H, 2.72; N, 14.27%.

[Rh^I(L)(CO)](NO_3) {[5](NO_3)}. A DMF solution of [**2**](NO_3) diffused by diethyl ether was standing for 1 week under a CO atmosphere (0.2 MPa) to give dark crystals, which were collected by filtration and dried under reduced pressure {yield 33% based on [**2**](NO_3)}. ESI-MS (in methanol): *m*/*z* 369.9 {[**5**]$^+$, relative intensity (*I*) = 100% in the range of *m*/*z* 200 to 2000}. ^{1}H NMR (600 MHz, in DMF-d_7, referenced to protio solvent impurities): δ 3.84 (s, 6H, C*H*$_3$), 7.69 (s, 2H, NC*H*), 7.82 (d, 2H, pyridine-*H*), 8.33 (s and t, 3H, NC*H* and pyridine-*H*).

Quantitative Analysis of H_2O_2 from the Reaction of 2 with O_2 by Iodometric Spectrophotometry. An aqueous solution of [**2**](NO_3) (50 μM, 10 mL), which was prepared from **1** with H_2, was stirred under an O_2 atmosphere (0.1 MPa) for 5 min. To the resulting solution was added $NaBPh_4$ (2.0 mg, 5.8 μmol) to precipitate Rh complexes. The precipitates were removed by filtration with filter paper. An excess amount of NaI (16 mg, 0.11 mmol) was added to the filtrate, and the resulting solution was monitored by UV-vis absorption spectroscopy. The yield of H_2O_2 based on [**2**](NO_3) was 53% calculated from the amount of I_3^- {yield = mol of I_3^-/ (mol of [**2**](NO_3)/2)}. The amount of I_3^- was estimated based on the UV-vis absorption spectrum ($\varepsilon = 2.4\times10^4$ M^{-1} cm^{-1} at 353 nm).[4]

Quantitative Analysis of H_2O_2 from the Reaction of 2 with O_2 by Using the Ti-TPyP Reaction Method. An aqueous solution of [**2**](NO_3) (50 μM, 10 mL), which was prepared from **1** with H_2, was stirred under an O_2 atmosphere (0.1 MPa) for 10 min. A portion (300 μL) of the resulting solution was diluted to 500 μL with water. To a portion (500 μL) of the resulting solution was added 4.8 M $HClO_4$ aqueous solution (500 μL) followed by the addition of 50 μM Ti-TPyP aqueous solution (500 μL) containing 50 mM HCl. After standing the resulting solution for 5 min, water (3.5 mL) was added to the solution, which was monitored by UV-vis absorption spectroscopy. The amount of H_2O_2 in the aqueous solution was determined by the change of absorbance at 433 nm.[5] The yield was determined to be 52% without CH_3COONa (50% with CH_3COONa) based on [**2**](NO_3) {yield = mol of H_2O_2/(mol of [**2**](NO_3)/2) according to the stoichiometry of eq 2}.

Catalytic Production of H_2O_2 with 1 under H_2 and O_2 Atmosphere. The amount of H_2O_2 formed was determined by using Ti-TPyP reagent, as monitored by UV-vis absorption spectroscopy. A polypropylene tube containing an aqueous CH_3COONa solution (0.5 M, 1.5 mL, pH 8.3) of [**1**]$(NO_3)_4$ (100 μM) was transferred to the reaction cylinder, and the reaction solution was stirred for 12 h under an H_2/O_2 (95/5) atmosphere at a pressure of 0.5, 0.9, 0.99, 1.5, or 1.9 MPa at 23 °C. After removing the H_2 and O_2 gases, the reaction solution was diluted 200 times. To a portion (500 μL) of the diluted solution was added 4.8 M $HClO_4$ aqueous solution (500 μL) followed by the addition of 50 μM Ti-TPyP aqueous solution (500 μL) containing 50 mM HCl. After standing the resulting solution for 5 min, water (3.5 mL) was added to the solution, which was monitored by UV-vis absorption spectroscopy. The amount of H_2O_2 in the aqueous solution was determined by the change of absorbance at 433 nm. The turnover numbers {turnover numbers (TONs) = mol of H_2O_2/mol of [**1**]$(NO_3)_4$} were determined as 298 (0.5 MPa), 433 (0.9 MPa), 476 (0.99 MPa), 676 (1.5 MPa), and 910 (1.9 MPa). The values of TON are the averages of at least three independent experiments. The TON was decreased to 51 without CH_3COONa. It was confirmed no production of H_2O_2 in the absence of **1**, H_2, or O_2. No nanoparticles were formed in the catalytic reaction, which was confirmed by DLS analysis. For the calculation of an initial rate of the catalytic reaction, the catalytic reaction was carried out for 1 h under an H_2/O_2 (95/5) atmosphere (1.9 MPa) at 23 °C. The formed H_2O_2 was quantified by the same method described above. The turnover frequency was calculated as 164 h^{-1} (163 mol kg_{cat}^{-1} h^{-1}). The pH of the catalytic solution was measured as 8.3 after the catalytic reaction.

Catalytic Production of H_2O_2 with 2 under H_2 and O_2 Atmosphere. The amount of H_2O_2 formed was determined by using Ti-TPyP reagent, as monitored by UV-vis absorption spectroscopy. A polypropylene tube containing an aqueous CH_3COONa solution (0.5 M, 1.5 mL) of [**1**]$(NO_3)_4$ (100 μM) was transferred to the reaction cylinder. The reaction solution was stirred for 3 h under an H_2 atmosphere (0.3 MPa) at 23 °C to generate **2** in situ. After removing H_2, the reaction solution was stirred for 12 h under an H_2/O_2 (95/5) atmosphere at a pressure of 1.9 MPa at 23 °C. After removing the H_2 and O_2 gases, the reaction solution was diluted 200 times. To a portion (500 μL) of the diluted solution was added 4.8 M $HClO_4$ aqueous solution (500 μL) followed by the addition of 50 μM Ti-TPyP aqueous solution (500 μL) containing 50 mM HCl. After standing the resulting solution for 5 min, water (3.5 mL) was added to the solution, which was monitored by UV-vis absorption spectroscopy. The

amount of H_2O_2 in the aqueous solution was determined by the change of absorbance at 433 nm. The TON {TON = mol of H_2O_2/mol of [**1**](NO_3)$_4$} was determined as 908.

The Initial Rate of the Catalytic H_2O_2 Production against the Catalyst Concentration. The catalytic reactions were carried out using [**1**](NO_3)$_4$ (1.1, 2.2, 3.0, 4.4, or 6.0 μM) for 30 min following the same procedure described above. The amount of H_2O_2 formed was determined by using Ti-TPyP reagent, as monitored by UV-vis absorption spectroscopy.

Qualitative Analysis of H_2O_2 Produced from the Catalytic Reaction with 1 by GC-MS. A polypropylene tube containing an aqueous CH_3COONa solution (0.5 M, 1.5 mL) of [**1**](NO_3)$_4$ (50 μM) was transferred to the reaction cylinder, and the reaction solution was stirred for 12 h under an H_2/O_2 (95/5) atmosphere (1.9 MPa) at 23 °C. After removing the H_2 and O_2 gases, the resulting solution was passed through the short silica gel column to remove Rh complex. The H_2O_2 in the resulting solution was detected by GC-MS.

Isotope Labeling Experiment for the $H_2{}^{18}O_2$ Production from the Catalytic Reaction with 1 Using H_2 and $^{18}O_2$. $^{18}O_2$ (50 mL) was injected to the Hiper glass cylinder containing an aqueous CH_3COONa solution (0.5 M, 1.5 mL) of [**1**](NO_3)$_4$ (50 μM), and the resulting solution was stirred for 12 h under an H_2 atmosphere (1.9 MPa) at 23 °C. After removing the H_2 and $^{18}O_2$ gases, the resulting solution was passed through the short silica gel column to remove Rh complex. The $H_2{}^{18}O_2$ in the resulting solution was detected by GC-MS.

Isotope Labeling Experiment for the Reaction of 2 with $H_2{}^{18}O_2$. An aqueous solution of $H_2{}^{18}O_2$ (10 μL) was added to an acetonitrile solution (500 μL) of [**2**](CF_3SO_3) (3.0 mg, 5.9 μmol), and the mixture solution was standing for 15 min. The resulting solution was passed through the short silica gel column to remove Rh complex. The produced $H_2{}^{18}O$ in the resulting solution was detected by GC-MS. The same reaction was carried out in a 4.5 mL polypropylene tube capped with a septum. The gas phase of the tube was sampled by a gas-tight syringe and monitored by GC-MS. There is no $^{18}O_2$ (m/z = 36) as shown in Figure S17.

Quantitative Analysis of $H_2{}^{18}O$ as a Decomposition Product from Reaction of H_2 with $^{18}O_2$ in $H_2{}^{16}O$. $^{18}O_2$ (60 mL) was injected to the Hiper glass cylinder containing an aqueous CH_3COONa solution (0.5 M, 1.5 mL) of [**1**](NO_3)$_4$ (100 μM), and the resulting solution was stirred for 12 h under an H_2 atmosphere (1.5 MPa) at 23 °C. After removing the

H_2 and $^{18}O_2$ gases, the resulting solution was passed through the short silica gel column to remove Rh complex. The $H_2{}^{18}O$ in the resulting solution was detected and quantified by using GC-MS. The amount of the formed $H_2{}^{18}O_2$ was determined by the Ti-TPyP reaction method. The yield of $H_2{}^{18}O$ was ca. 10% based on the formed $H_2{}^{18}O_2$ (the yield of $H_2{}^{18}O$ is the average of three independent experiments. yield = mol of $H_2{}^{18}O$/mol of $H_2{}^{18}O_2$).

Titration Experiments of 1 or 2 with CH_3COONa. Spectrophotometric titrations of an aqueous solution of [**1**]$(NO_3)_4$ (1.26 mM, pH 3.9) or [**2**](NO_3) (1.26 mM, pH 4.9) were conducted using an aqueous solution of CH_3COONa under an N_2 atmosphere, as monitored by the absorption at 400 nm for **1** or 514 nm for **2**.

Electrochemical Analysis. Electrochemical measurements were conducted in an aqueous solution of [**3**]$(NO_3)_3$ or [**6**]$(NO_3)_3$ with CH_3COONa (0.1 M) as a supporting electrolyte on a BAS660A electrochemical analyzer using a carbon working electrode, a Pt counter electrode, and an Ag/AgCl reference electrode at room temperature.

X-ray Crystallographic Analyses of CH_3CN-coordinated Rh^{II} Dimer Complex 4 and CO-coordinated Rh^{I} Complex 5. A single crystal of CH_3CN-coordinated **1** $[Rh^{II}_2(L)_2(OH_2)_2(CH_3CN)_2](CF_3SO_3)_4$ {[**4**]$(CF_3SO_3)_4$} was obtained by the slow evaporation of the aqueous solution of **1** with a few drops of acetonitrile at 23 °C after replacing the NO_3^- ions with $CF_3SO_3^-$ ions. A single crystal of CO-coordinated **2** $[Rh^{I}(L)(CO)](NO_3)$ {[**5**](NO_3)} was obtained from the DMF solution of [**2**](NO_3) diffused with diethyl ether under a CO atmosphere (0.2 MPa). Measurements were made on a Rigaku XtaLAB P200 with confocal monochromated Mo-Kα radiation (λ = 0.7107 Å). Data were collected and processed using the CrystalClear program. All calculations were performed using the CrystalStructure crystallographic software package and the program Olex 2. Crystallographic data for **4** and **5** have been deposited with the Cambridge Crystallographic Data Centre as Supplementary Publication Nos. 2177260 (**4**) and 2177261 (**5**). Copies of the data can be obtained free of charge on application to CCDC, 12 Union Road, Cambridge CB2 1EZ, UK {fax: (+44)1223-336-033; e-mail: deposit@ccdc.cam.ac.uk}.

Table S1. Characterization of Rh complexes **1**, **2**, **3**, **4**, and **5**

	**1**	**2**	**3**	**4**	**5**
X-ray	–	–	–	Figure 1	Figure S11
ESI-MS	–	–	–	–	Figure S12
^{1}H NMR	Figure S4	Figure S10	Figure S1	–	Figure S13
^{13}C NMR	Figure S5	–	Figure S2	–	–
C–H COSY	Figure S6	–	Figure S3	–	–
UV-vis-NIR	Figure S7	Figure S9	–	–	–
XPS	Figure S8	Figure S8	Figure S8	–	–

Table S2. Production cost of 1 kg of H_2O_2 by anthraquinone process (50,000 t/year plant scale) and production cost of 1 g of H_2O_2 by this method (lab scale)

Entry		Production cost (U.S. $) of 1 kg of H_2O_2 by anthraquinone process (50,000 t/year plant scale)[6,a] U.S. $/kg	Production cost (U.S. $) of 1 g of H_2O_2 by this method (lab scale)[b] U.S. $/g
1	Pd catalyst	0.021	−
2	Rh catalyst	−	1.37
3	anthraquinone	0.0053	−
4	H_2	0.13	0.0076
5	O_2	0	0.0015
6	solvent	0.0071	0.00076
7	electricity	0.069	0.0053
8	vapor	0.058	0
9	cooling water	0.051	0
10	transport cost	0.11	0
	total	0.45	1.4

[a]Cost to build a 50,000 t/year plant: U.S. $115,000,000. [b]Cost of the high-pressure glass cylinder: U.S. $1,900 (131 JPY/USD).

Table S3. Structures and bond distances of the unsupported Rh^{II} dimer complexes

Entry	Structure[a]	Rh–Rh distance (Å)	Ref
1	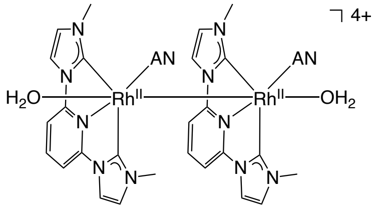	2.7378(4)	this work
2	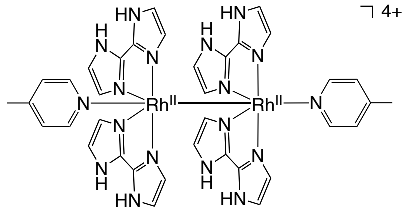	2.7052(5)	7
3	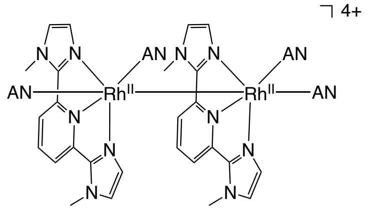	2.6710(8)	2

[a]acetonitrile (AN)

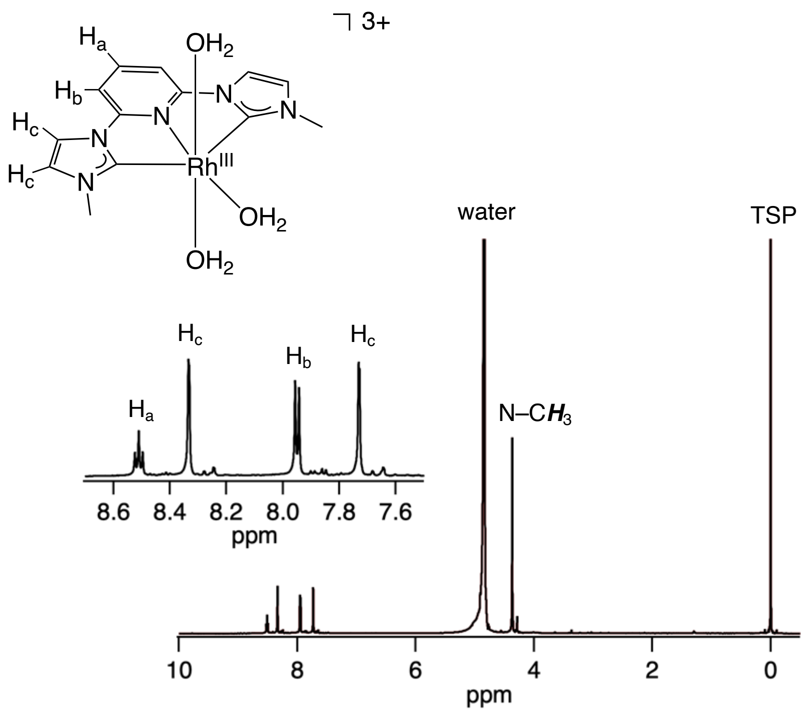

Figure S1. A ^{1}H NMR spectrum of **3** in D_2O. Inset: the ^{1}H NMR spectrum enlarged in the aromatic region between 7.5 and 8.7 ppm.

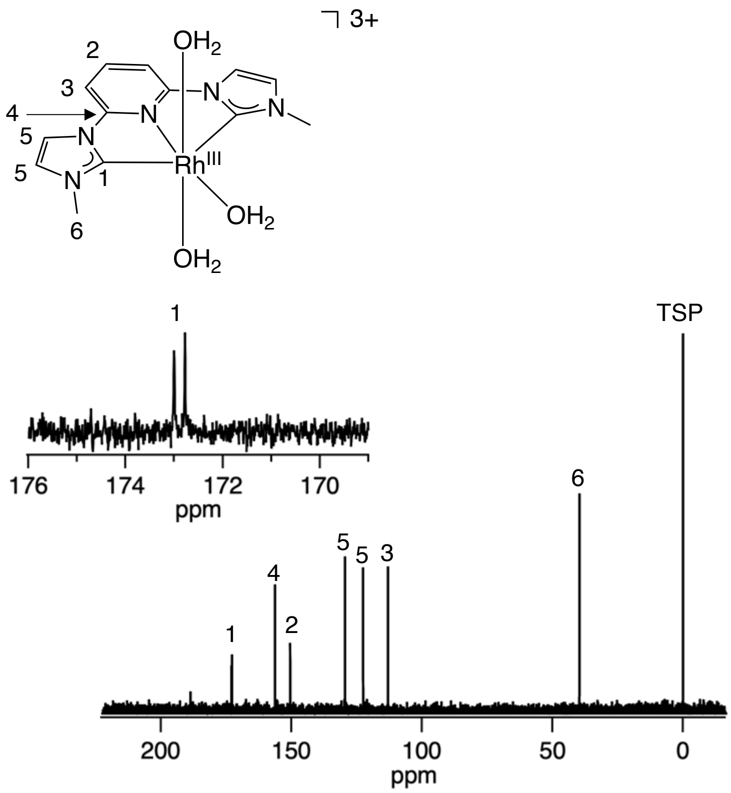

Figure S2. A ^{13}C NMR spectrum of **3** in D_2O. Inset: the enlarged ^{13}C NMR spectrum between 169 and 176 ppm.

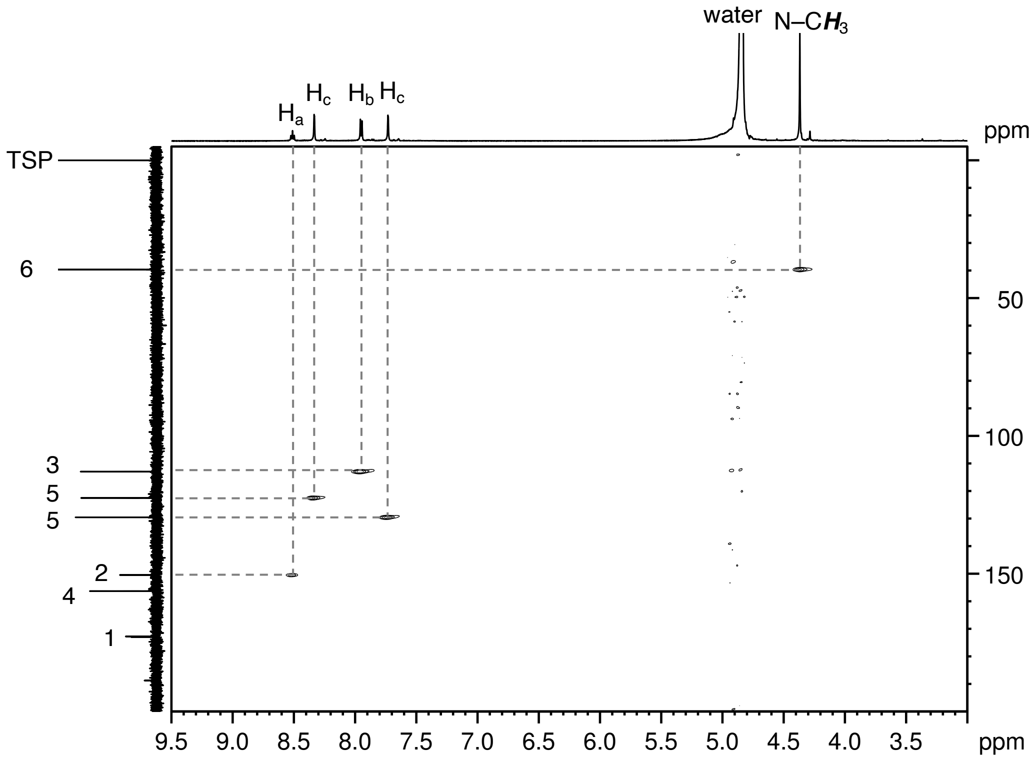

Figure S3. A C–H COSY spectrum of **3** in D_2O.

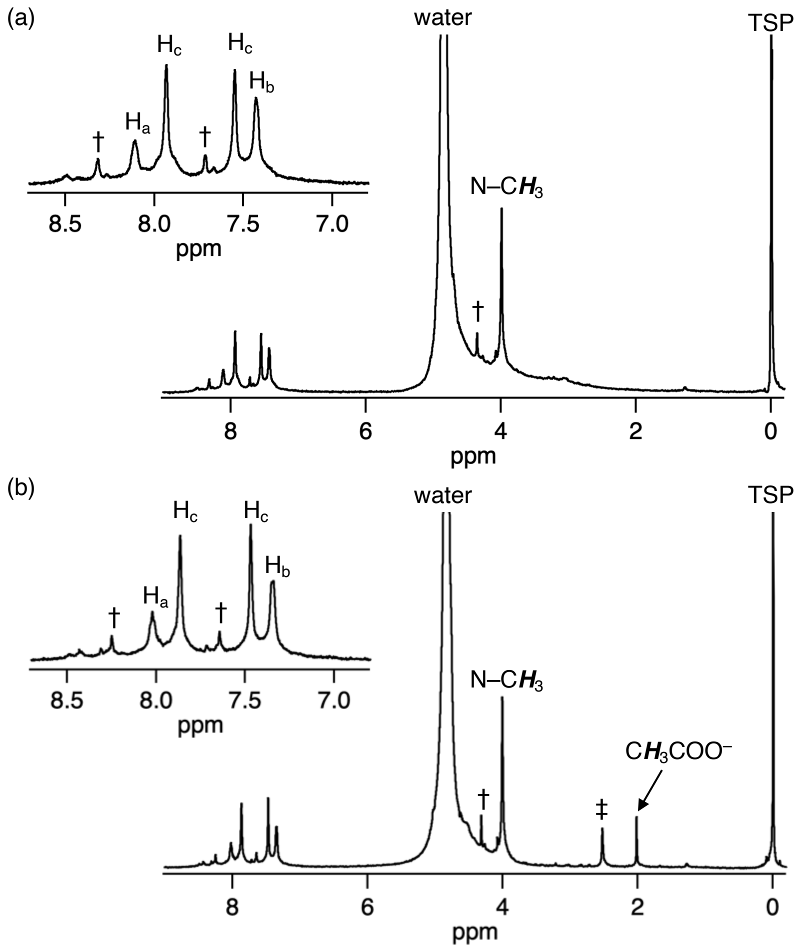

Figure S4. (a) A ^{1}H NMR spectrum of **1** in D_2O. Inset: the ^{1}H NMR spectrum enlarged in the aromatic region between 6.8 and 8.7 ppm. †: Not assigned peaks. (b) A ^{1}H NMR spectrum of **1** with CH_3COONa (2 eq) in D_2O. Inset: the ^{1}H NMR spectrum enlarged in the aromatic region between 6.8 and 8.7 ppm. †: Not assigned peaks. ‡: Acetate ion interacted with complex **1** in a 1:1 ratio, calculated from the integration of the peak. The labels of hydrogen atoms, H_a, H_b, and H_c, are shown in Figure S1.

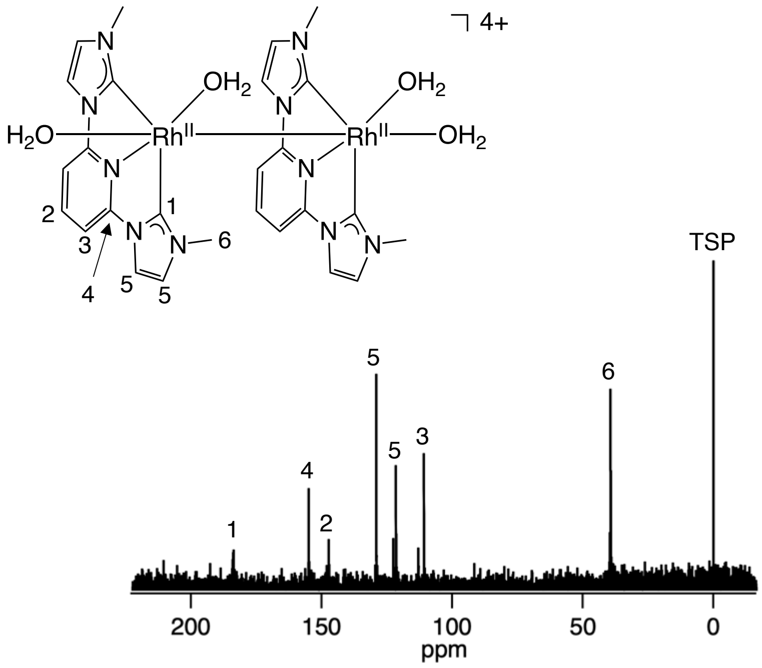

Figure S5. A ^{13}C NMR spectrum of **1** in D_2O.

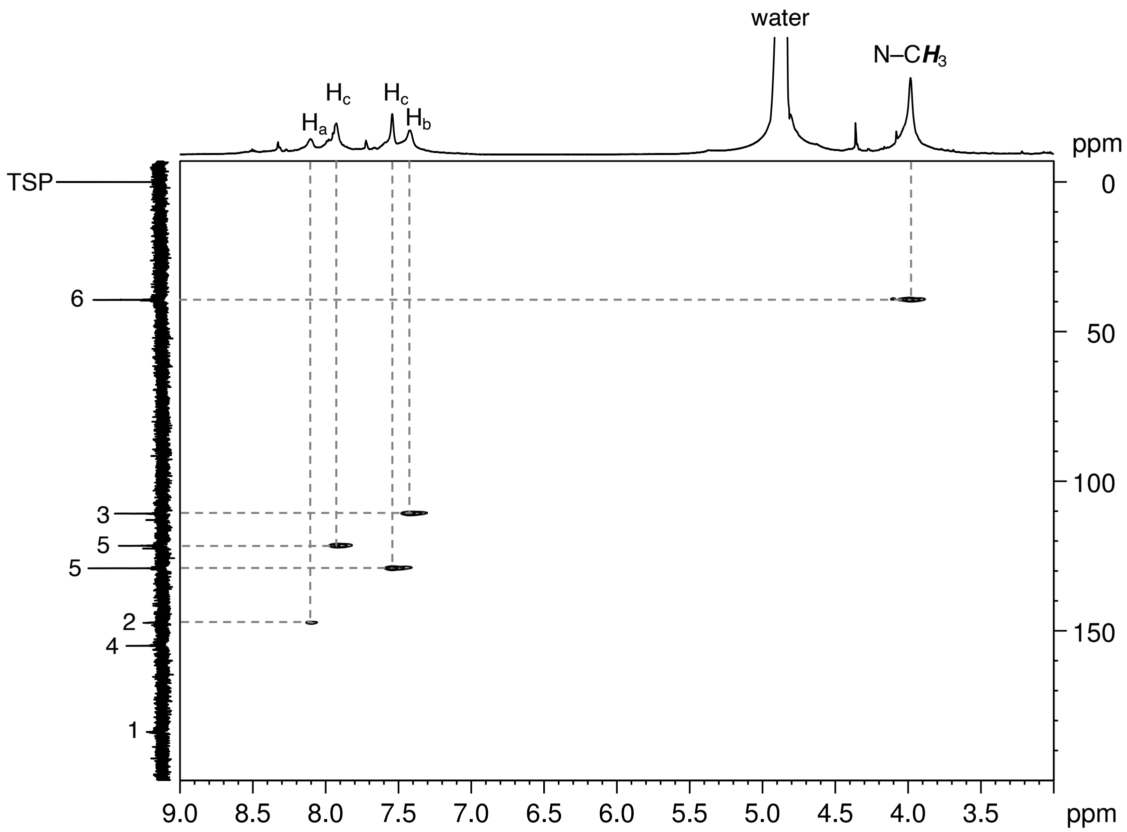

Figure S6. A C–H COSY spectrum of **1** in D_2O.

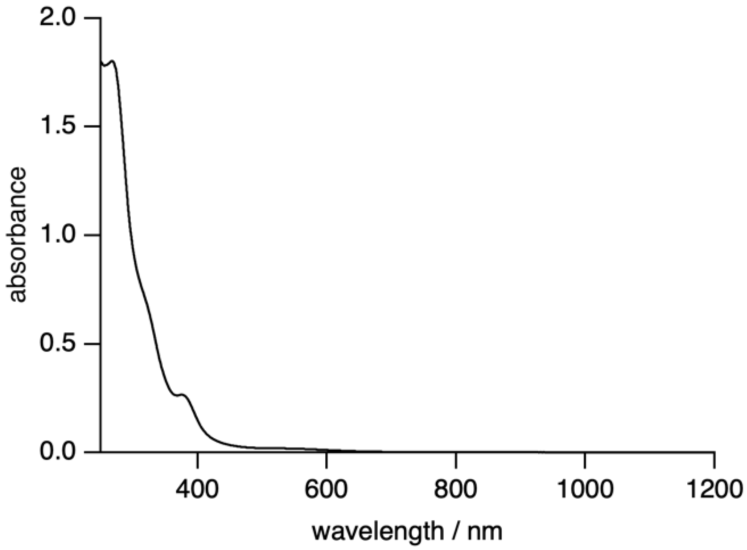

Figure S7. A UV-vis-NIR absorption spectrum of **1** (0.63 mM) in water. The light path length is 0.1 cm.

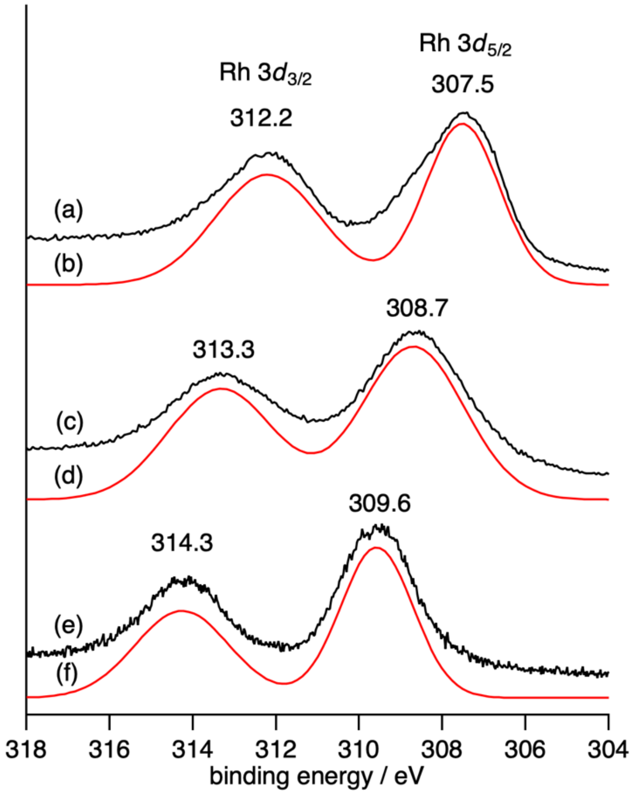

Figure S8. XPS (X-ray photoelectron spectroscopy) spectra in the Rh 3d region for (a) Rh^{I} complex **2**, (c) Rh^{II} complex **1**, and (e) Rh^{III} complex **3**. XPS fitting for (b) **2**, (d) **1**, and (f) **3**.

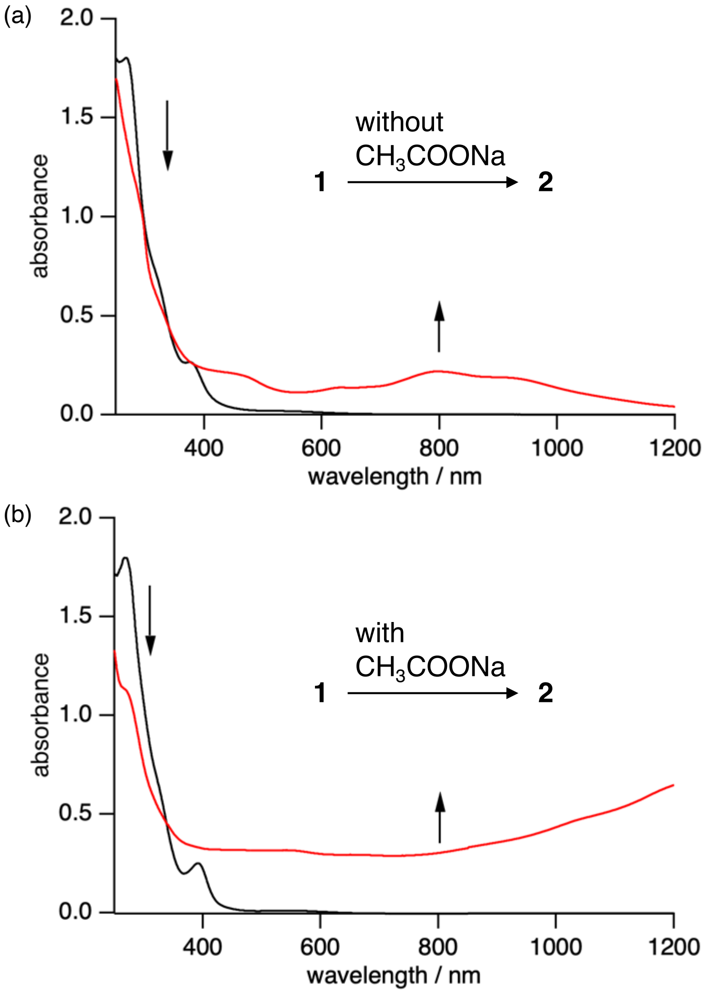

Figure S9. (a) Under stoichiometric conditions, UV-vis-NIR absorption spectra of complex **1** (0.63 mM) in water (black line) and complex **2** prepared from the reaction of **1** (0.63 mM) with H_2 (0.5 MPa) (red line) at 23 °C for 2 h in water at pH 3.9. (b) Under stoichiometric conditions, UV-vis-NIR absorption spectra of complex **1** (0.63 mM) in water with CH_3COONa (1.26 mM) (black line) and complex **2** prepared from the reaction of **1** (0.63 mM) with H_2 (0.5 MPa) (red line) at 23 °C for 2 h in water with CH_3COONa (1.26 mM) at pH 4.9. The light path length is 0.1 cm.

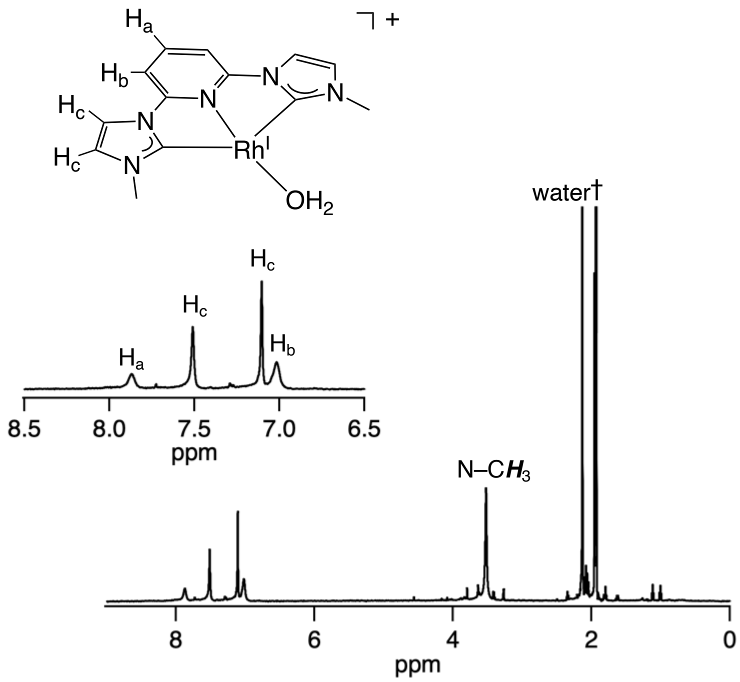

Figure S10. A ^{1}H NMR spectrum of **2** in CD_3CN. Inset: the ^{1}H NMR spectrum enlarged in the aromatic region between 6.5 and 8.5 ppm. †: Peak of acetonitrile.

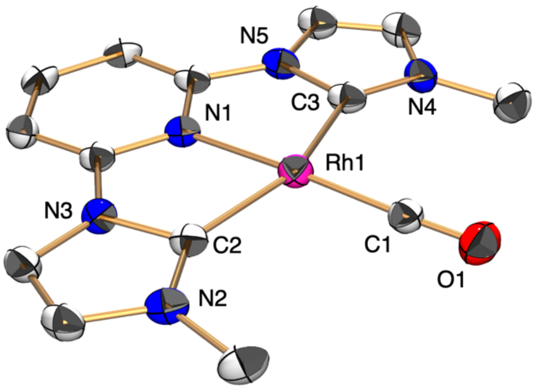

Figure S11. An ORTEP drawing of CO-coordinated **2** [Rh^{I}(L)(CO)](NO_3) {[**5**](NO_3)} with the ellipsoids at 50% probability. Counteranion (NO_3^-) and hydrogen atoms are omitted for clarity. L = 2,6-bis(1-methylimidazol-2-ylidene)pyridine.

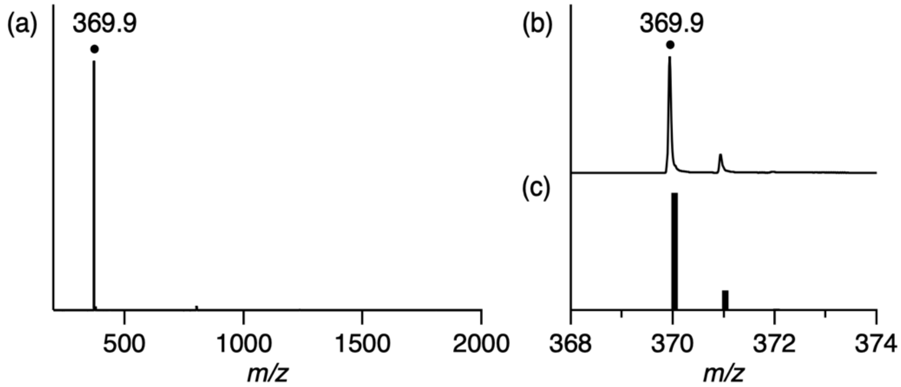

Figure S12. (a) A positive-ion ESI mass spectrum of **5** in methanol. The signal at *m*/*z* 369.9 corresponds to [**5**]$^+$. (b) The signal at *m*/*z* 369.9 for [**5**]$^+$. (c) The calculated isotopic distribution for [**5**]$^+$.

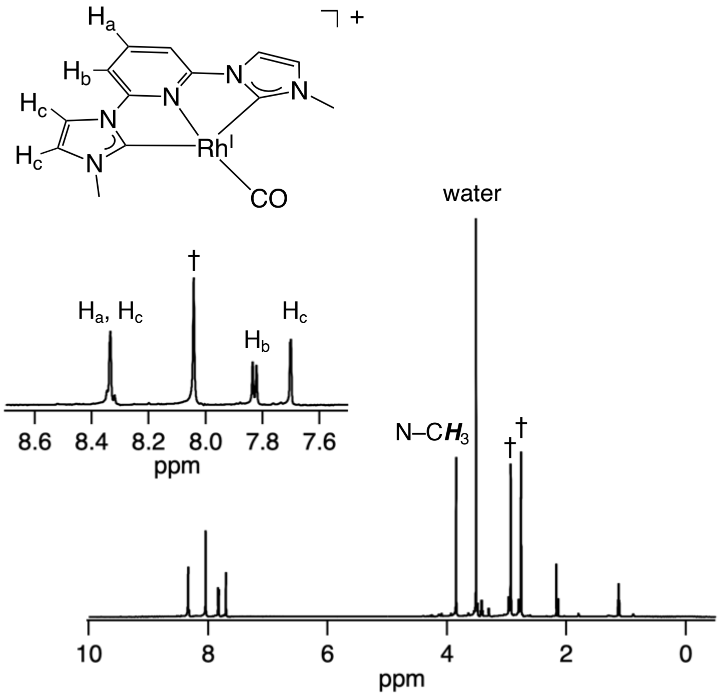

Figure S13. A ^{1}H NMR spectrum of **5** in DMF-d_7. Inset: the ^{1}H NMR spectrum enlarged in the aromatic region between 7.5 and 8.7 ppm. †: Peaks of DMF.

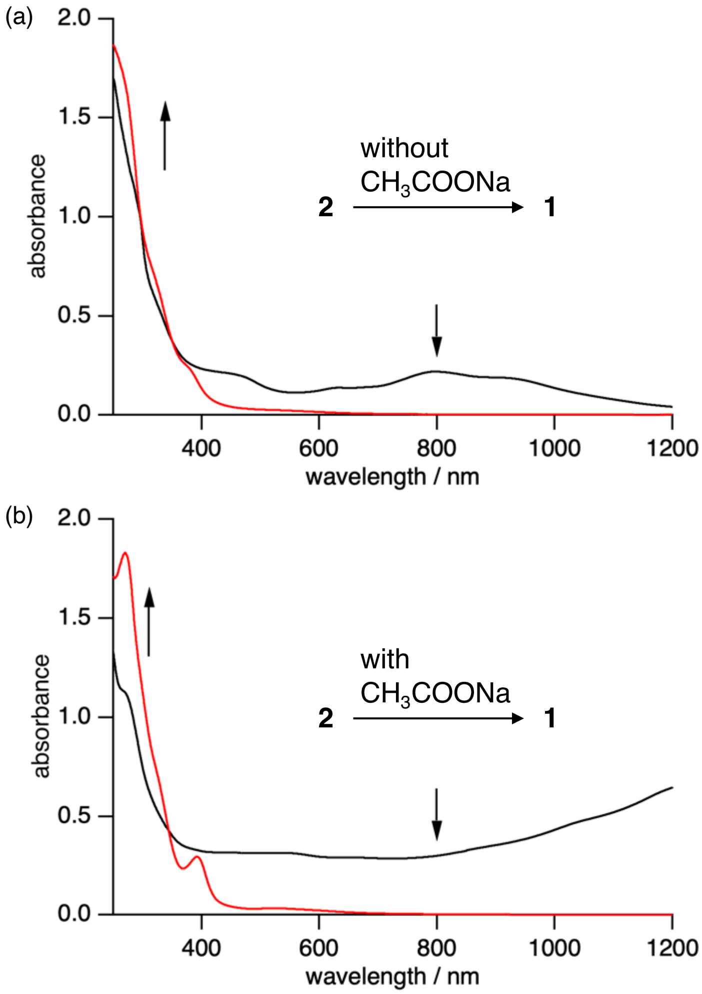

Figure S14. (a) Under stoichiometric conditions, UV-vis-NIR absorption spectra of complex **2** (1.25 mM) in water (black line) and the reaction of complex **2** (1.25 mM) with O_2 (1.0 mL) (red line) at 23 °C for 5 min in water at pH 3.9. (b) Under stoichiometric conditions, UV-vis-NIR absorption spectra of complex **2** (1.25 mM) in water with CH_3COONa (1.26 mM) (black line) and the reaction of complex **2** (1.25 mM) with O_2 (1.0 mL) (red line) at 23 °C for 5 min in water with CH_3COONa (1.26 mM) at pH 4.9. The light path length is 0.1 cm.

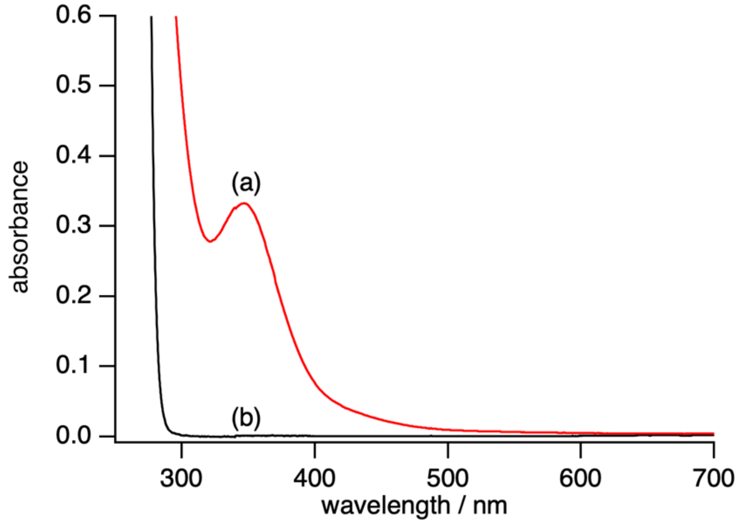

Figure S15. (a) Quantitative analysis of H_2O_2 from the reaction of complex **2** (50 μM) with O_2 (3.0 mL) by iodometric spectrophotometry using NaI (16 mg, 0.11 mmol) as the redox indicator. (b) As for (a) but without complex **2**.

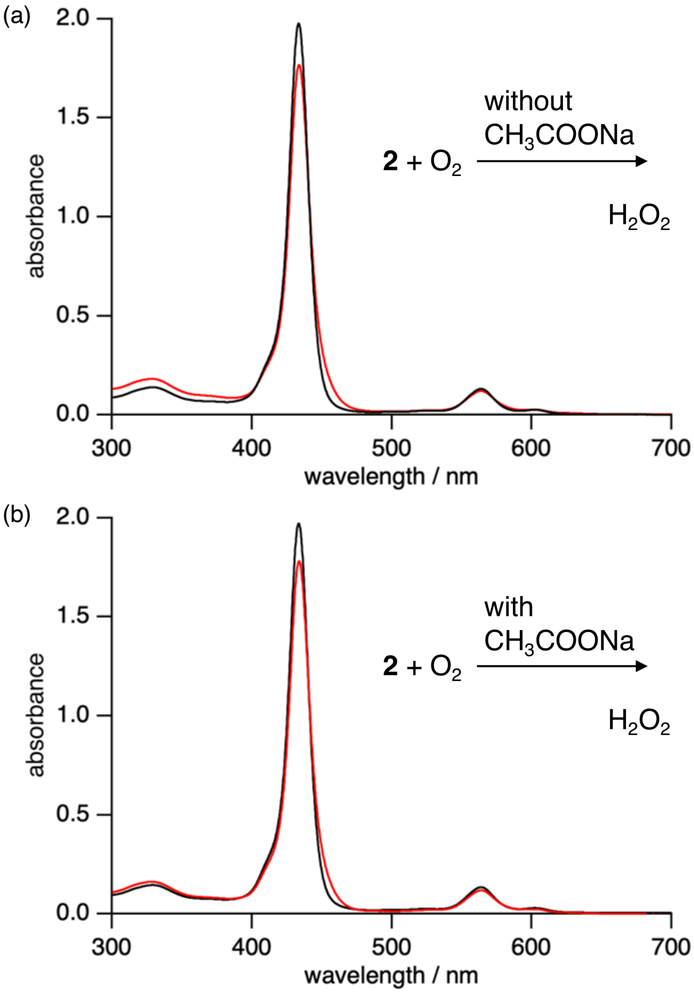

Figure S16. (a) Under stoichiometric conditions, quantitative analysis of H_2O_2 from the reaction of complex **2** (50 μM) with O_2 (3.0 mL) by the titration with oxo[5,10,15,20-tetra(4-pyridyl)porphyrinato]titanium(IV) complex (red line) and the control experiment without complex **2** (black line). (b) Under stoichiometric conditions, quantitative analysis of H_2O_2 from the reaction of complex **2** (50 μM) with O_2 (3.0 mL) by the titration with oxo[5,10,15,20-tetra(4-pyridyl)porphyrinato]titanium(IV) complex (red line) and the control experiment without complex **2** (black line) in the presence of CH_3COONa (0.5 M).

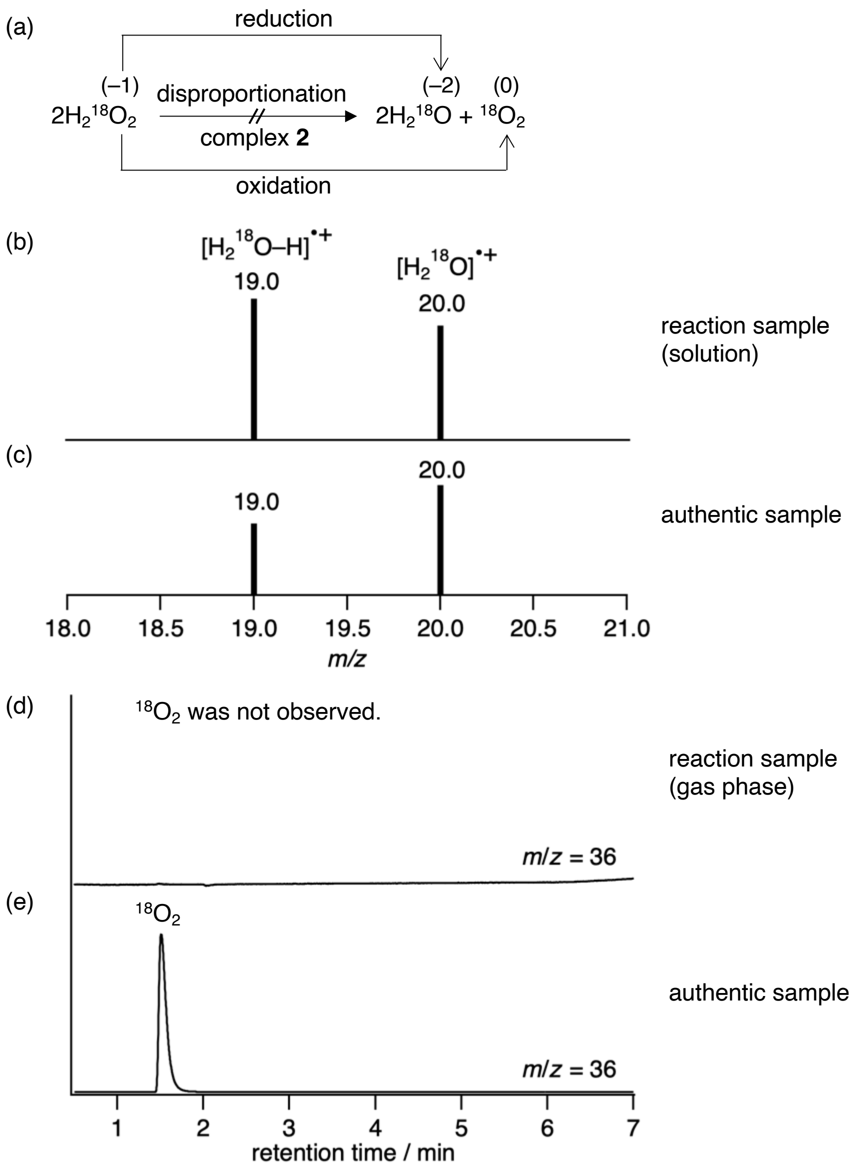

Figure S17. The produced H_2O_2 decomposed less than 10% into H_2O, which was confirmed by the isotope labeling experiment using $H_2{}^{18}O_2$. (a) Complex **2** (11.8 mM) reacts with $H_2{}^{18}O_2$ (10 µL) in CH_3CN but does not generate $^{18}O_2$ (no disproportionation occurs). (b) A positive-ion GC-MS analysis of the solution. (c) Authentic $H_2{}^{18}O$. (d) Extracted ion chromatograms of GC mass analysis of the gas phase of the reaction solution. (e) Authentic $^{18}O_2$.

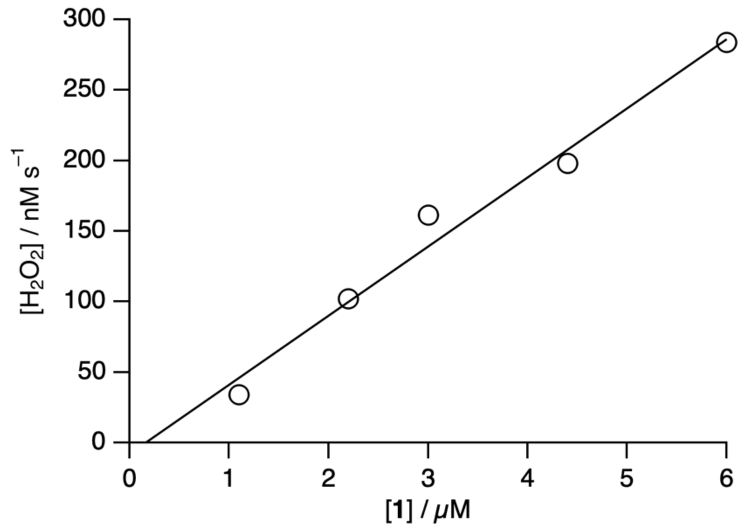

Figure S18. A plot of initial rate of catalytic H_2O_2 production against the concentration of **1** (1.1–6.0 μM) in the catalytic reaction of **1** with H_2/O_2 (95/5, 1.9 MPa) at 23 °C. The initial rate of H_2O_2 production was determined for 1800 s.

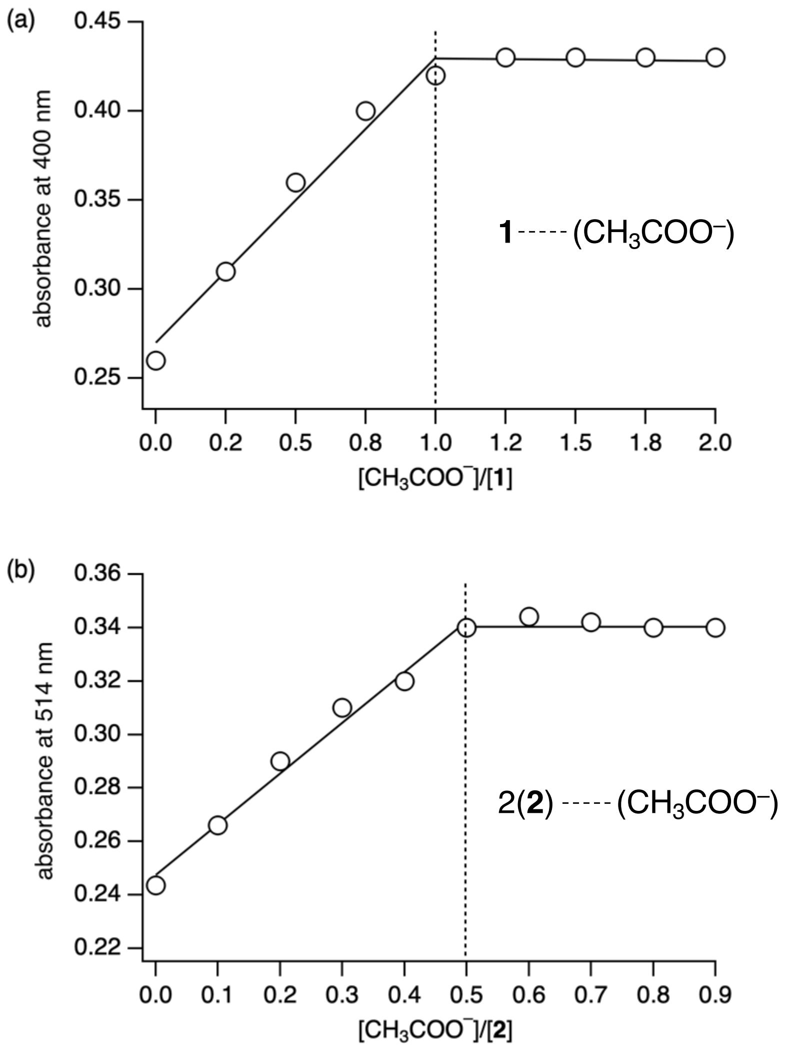

Figure S19. (a) Spectrophotometric titration of an aqueous solution of **1** (1.26 mM, pH 3.9) with an aqueous solution of CH_3COONa under an N_2 atmosphere, as monitored by the absorption at 400 nm. (b) Spectrophotometric titration of an aqueous solution of **2** (1.26 mM, pH 4.9) with an aqueous solution of CH_3COONa under an N_2 atmosphere, as monitored by the absorption at 514 nm.

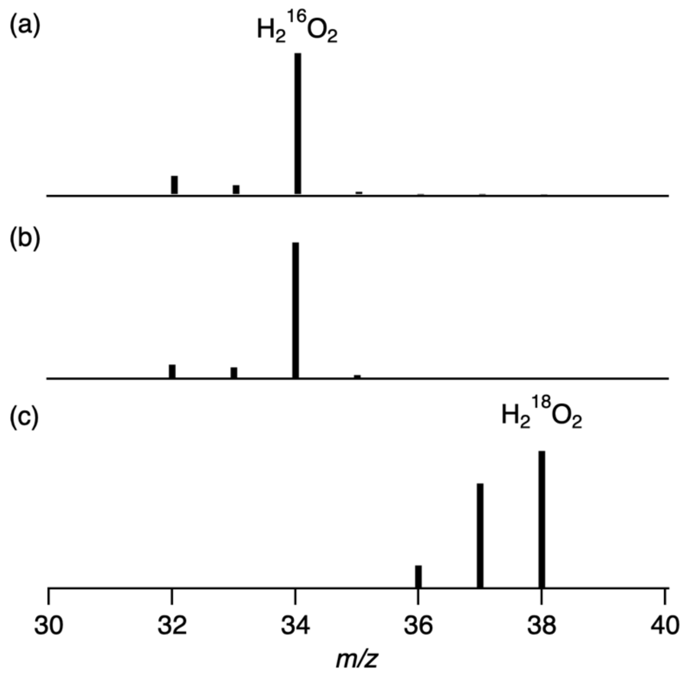

Figure S20. (a) A positive-ion GC mass spectrum of $H_2{}^{16}O_2$ obtained from the catalytic reaction of complex **1** (50 μM) in an aqueous CH_3COONa solution (1.5 mL) with H_2 and O_2. (b) A positive-ion GC mass spectrum of authentic $H_2{}^{16}O_2$. (c) A positive-ion GC mass spectrum of $H_2{}^{18}O_2$ obtained from the catalytic reaction of complex **1** (50 μM) in an aqueous CH_3COONa solution (1.5 mL) with H_2 and $^{18}O_2$. The detailed procedures are described in the experimental section.

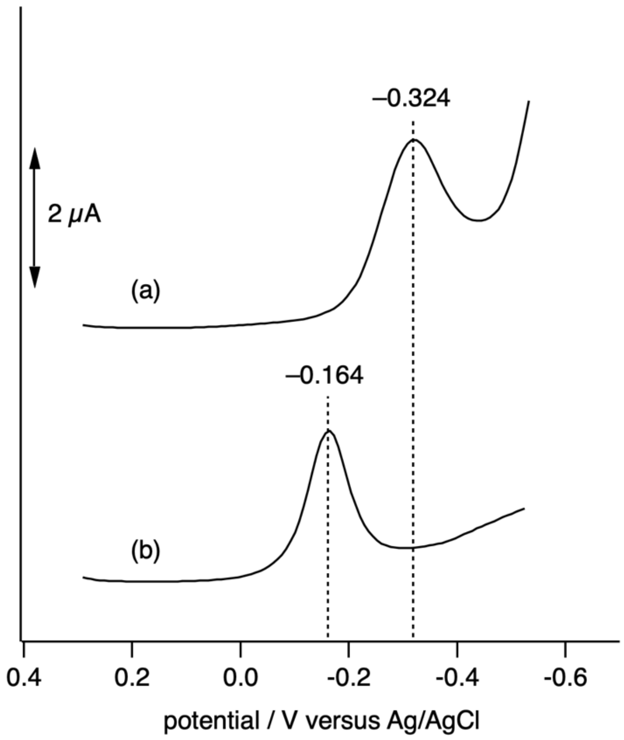

Figure S21. Differential pulse voltammograms of (a) complex **3** and (b) complex **6** in an aqueous solution of CH_3COONa (0.1 M) (complex concentration: 2.5 mM).

References

(1) Frey, G. D.; Rentzsch, C. F.; von Preysing, D.; Scherg, T.; Mühlhofer, M.; Herdtweck, E.; Herrmann, W. A. Rhodium and iridium complexes of N-heterocyclic carbenes: Structural investigations and their catalytic properties in the borylation reaction. *J. Organomet. Chem.* **2006**, *691*, 5725–5738.

(2) Ogo, S.; Minh, L. T. T.; Kikunaga, T.; Ando, T.; Matsumoto, T.; Yatabe, T.; Kato, K. Direct Synthesis of Hydrogen Peroxide in Water by Means of a Rh-Based Catalyst. *Organometallics* **2020**, *39*, 3731–3741.

(3) Moulder, J. F.; Stickle, W. F.; Sobol, P. E.; Bomben, K. D. In *Handbook of X-ray Photoelectron Spectroscopy*, J. Chastin, R. C. Jr. King, Eds., Physical Electronics, Inc.: Eden Prairie, MN, **1995**, pp 40–41.

(4) Gobert, S. R. L.; Kuhn, S.; Braeken, L.; Thomassen, L. C. J. Characterization of Milli- and Microflow Reactors: Mixing Efficiency and Residence Time Distribution. *Org. Process Res. Dev.* **2017**, *21*, 531–542.

(5) Matsubara, C.; Kawamoto, N.; Takamura, K. Oxo[5,10,15,20-tetra(4-pyridyl)porphyrinato]titanium(IV): An Ultra-High Sensitivity Spectrophotometric Reagent for Hydrogen Peroxide. *Analyst* **1992**, *117*, 1781−1784.

(6) Nexant, CHEMSYSTEMS PERP PROGRAM PERP 07/08-3 Hydrogen Peroxide **2009**, 65.

(7) Long, J.; Uemura, K.; Ebihara, M. Assembled structures of tetrakis(biimidazole)dirhodium complexes hydrogen-bonded with common inorganic anions. *Acta Crystallogr. B* **2014**, *70*, 1006–1019.
